# On lifestyle trends, health and mosquitoes: Formulating welfare levels for control of the Asian tiger mosquito in Greece

**DOI:** 10.1371/journal.pntd.0007467

**Published:** 2019-06-04

**Authors:** Antonios Kolimenakis, Kostas Bithas, Dionysis Latinopoulos, Clive Richardson

**Affiliations:** 1 Institute of Urban Environment & Human Resources, Department of Economic and Regional Development, Panteion University, Kallithea, Athens, Greece; 2 School of Spatial Planning and Development, Aristotle University of Thessaloniki, Greece; University of Heidelberg, GERMANY

## Abstract

The expansion of urban ecosystems and climate change, both outcomes of massive lifestyle changes, contribute to a series of side effects such as environmental deterioration, spread of diseases, increased greenhouse gas emissions and introduction of invasive species. In the case of the Athens metropolitan area, an invasive mosquito species—the Asian tiger mosquito (*Aedes albopictus*)–has spread widely in the last decade. This spread is favoured within urban environments and is also affected by changing climatic trends. The Asian tiger mosquito is accompanied by risks of mosquito-borne diseases, greater nuisance levels, and increased expenses incurring for its confrontation. The main aims of this paper are (i) to estimate the various costs associated with the control of this invasive species, as well as its health and nuisance impacts, (ii) to evaluate the level of citizens’ well-being from averting these impacts and (iii) to record citizens’ and experts’ perceptions regarding alternative control measures. Evidence shows that experts tend to place a high value on mosquito control when associated with serious health risks, while citizens are more sensitive and concerned about the environmental impacts of control methods. The synthesis of results produced by the current study could act as a preliminary guide for the estimation of societal welfare from the confrontation of similar problems in the context of a complex ecosystem.

## Author summary

This paper is based on several years’ collaboration among researchers from various disciplines, key health policy makers and stakeholders in an attempt to evaluate the economic dimensions related to the presence of the Asian Tiger Mosquito (*Aedes albopictus*) and the challenges of tackling mosquito-borne disease outbreaks in Greece and Southern Europe. Similar studies have been conducted and continue to be published in Europe and the USA examining the socioeconomic benefit from the implementation of relevant control and prevention strategies. These studies conclude that there are significant benefits related both to the reduction of nuisance levels and the reduction of the health risks posed by various mosquito species. In our case, the application of an updated economic analysis on the effectiveness of relevant public control and prevention programs provides essential information for public health decision-making, bearing in mind the significant restructuring of the public sector and the fiscal crisis apparent in the European South.

## Introduction

Recent reports highlight the impacts and risks to human and natural systems linked to global warming of 1.5°C compared to temperatures in the pre-industrial period [1]. The implications of rising temperatures for human health around the globe include changes in disease vector survival and pathogen development, and the emerging new sanitary and environmental risks are directly related to various socioeconomic impacts. Recent studies indicate that intense urbanization favours the spread of vector-borne diseases, which may also flourish due to the higher density of both people and animals (both domestic and peridomestic ones), as well as due to various environmental and socioeconomic modifications [2–4]. In addition, the globalization of trade and travel has facilitated the spread and establishment of invasive alien species (IAS). Insects predominate among non-native terrestrial invertebrates in Europe: of 1,522 established species, 1,306 (86%) are insects [5]. The IAS inadvertently introduced into Europe include several invasive mosquito species (IMS), which have found environmental and climatic conditions favourable for the establishment of permanent populations. These IMS are recognised as responsible for the emergence or reappearance of mosquito-borne diseases such as chikungunya, dengue and West Nile virus (WNV).

One IMS of major public health concern in Southern Europe is *Aedes albopictus*, the Asian tiger mosquito, which arrived in Europe in Albania in 1979 and then Italy in the early 1990s, through the trade in used tires. *Ae*. *albopictus* is already established in large areas of Greece and Southern Europe [6–8] and studies indicate that its rate of expansion in Greece is quite rapid [9–11]. *Ae*. *albopictus* has already been responsible for transmitting both dengue and chikungunya viruses in continental Europe, including over 200 laboratory-confirmed cases of the latter in Italy (Region of Emilia Romagna) in 2007 [12,13] and local dengue transmission in Croatia and France [14,15].

The IMS problem may affect the economy and society in various ways, through impacts on human and animal health, as well as on various services and activities. These impacts generate certain economic costs related to control strategies, public health measures, treatment of illness, productivity losses, information and awareness campaigns, and losses in tourism and other sectors. Economic impacts can be direct or indirect. Direct economic impacts are usually expressed as the net increase in public health spending as a result of the appearance of IMS and include, among other things, control-and-surveillance programs, private expenditures and direct medical costs. Direct impacts are the most clearly defined impacts as they can be explicitly expressed in monetary values. On the other hand, indirect impacts include the costs associated with new research and management services (in both the public and private sectors of the economy), as well as the effects of IMS on tourism, etc [16–19].

Thus, the gradual establishment of higher IMS populations in Greece has been accompanied by greater risks of mosquito-borne diseases, increased costs of implementing prevention measures, higher nuisance levels and side-effects on tourism and other economic sectors. The aim of this paper is thus to present the main categories of costs related to the aforementioned problem, to evaluate the potential benefit of enhanced prevention measures and to examine citizens’ and experts’ opinions concerning the various socioeconomic aspects of the problem. In this framework, the present study offers a chance to consider the evaluation and selection of strategies for similar socio-ecological problems, by various interest groups, under the prism of different institutional approaches in an ecosystemic context.

## Methods

Prevention and control costs and data on health impacts were collected and analyzed in collaboration with the National Public Health Organization (formerly known as the Hellenic Centre for Disease Control and Prevention (HCDCP)), public health agencies and private companies specializing in mosquito control activities. In a previous work, a Cost-of-Illness study was carried out to estimate medical costs and productivity losses, from the West Nile Virus (2010) [20] while recent estimates are presented here concerning medical costs incurred by imported cases of dengue, chikungunya and Zika virus in Greece for the years 2013–2017. Citizens’ willingness to pay (WTP) for improved mosquito control programs was also based on an earlier study, which employed a contingent valuation method (CVM), specifically the discrete choice method [21].

**Fig 1:**
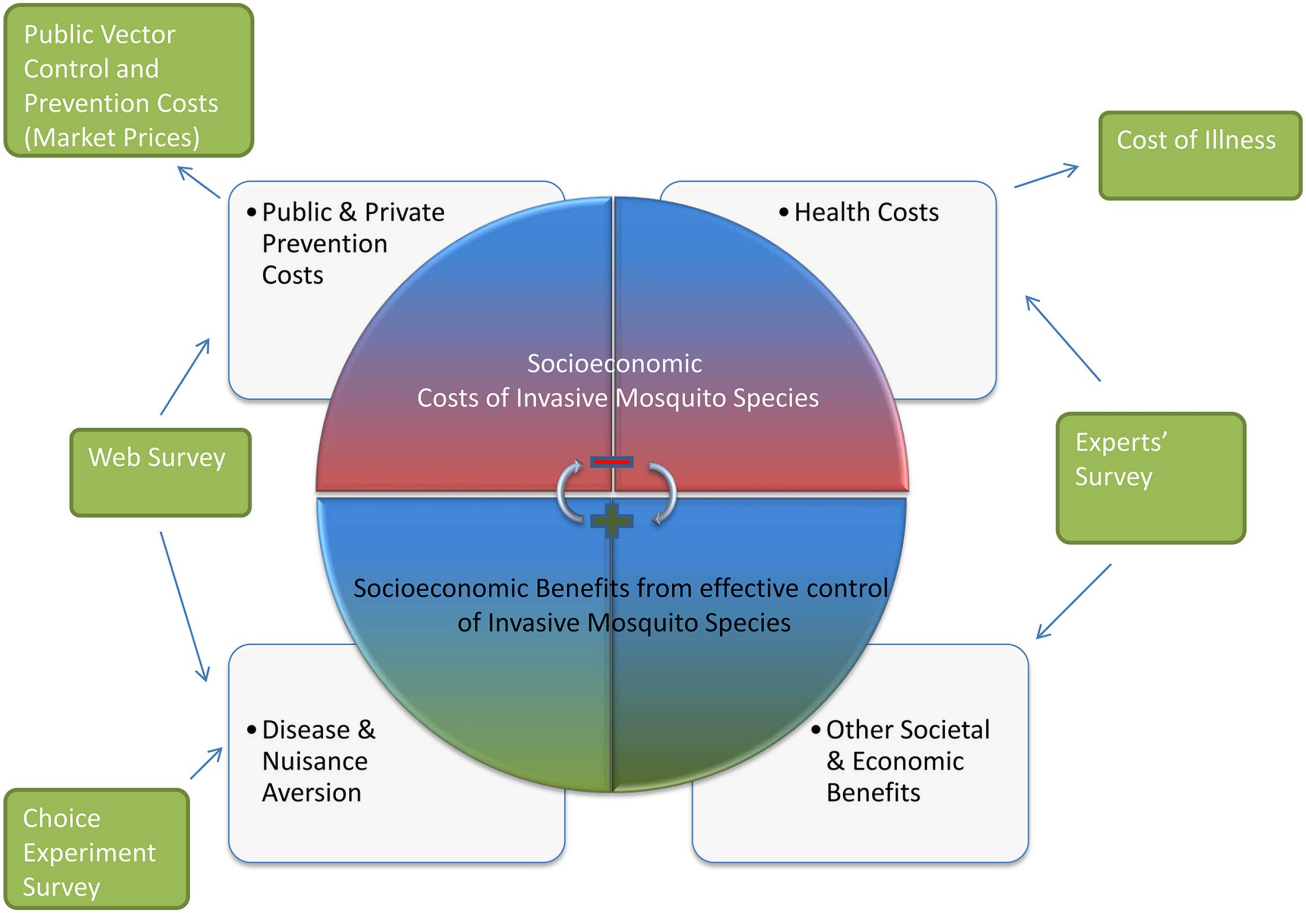
The different methods employed for the estimation of socioeconomic costs and benefits associated with the problem of invasive mosquito species.

Two new surveys were conducted for the present paper. These provide a deeper exploration of the socioeconomic impacts and benefits of implementing improved prevention and control strategies. The first was a nationwide web-based survey aiming to record citizens’ opinions and attitudes, and the second was a small-scale survey of experts involved in mosquito control activities in Greece. Fig 1 presents all the methods implemented and how they contribute to the overall estimation of the identified costs and benefits associated with the problem of IMS. It should be pointed out that costs and benefits are somehow interrelated, so that the elimination of the socioeconomic costs entails a positive consequence on the benefit side resulting from the control of IMS.

### Estimation of public control and prevention costs

The annual public control and prevention costs examined in this study consist mainly of: (a) annual mosquito vector control activities, (b) contingency costs incurred in response to the WNV epidemic by the responsible national agency, the National Public Health Organization (NPHO), and (c) costs of the additional screening of blood donations that is imposed because of the risk of transmission of WNV through blood transfusion. Market prices for vector control activities were provided directly by regional and municipal authorities and private companies. The annual costs incurred by the NPHO from 2010 to 2013 were extracted from official reports and databases. The cost of additional blood safety testing was provided by the Hellenic National Blood Centre.

### Estimation of health impact costs

Health impact costs were assessed in two ways. First of all, we estimated the medical costs for all imported cases of dengue, chikungunya and Zika virus in Greece for the period 2013–2017 (Table 1). This calculation was based on anonymized data on the duration of hospitalization of reported cases, including intensive care treatment, provided through the official records of NPHO. It should be noted that the reported cases consisted mainly of infected travelers from chikungunya, dengue and Zika endemic countries who presented symptoms of these diseases upon their return to Greece. These estimates of medical costs are similar to those in the recent literature regarding the imported chikungunya cases in Italy (based on 2015 data), and the 2005–2006 chikungunya epidemic in La Reunion [22, 23].

**Table 1:** Cost of illness for reported imported cases of Dengue, Chikungunya and Zika virus in Greece for the period 2013–2017.

Infection	Year	Hospitalization days (non ICU*)	Days in ICU*	Hospitalization cost	Additional hospitalization cost in ICU	Productivity losses (during hospitalization)	Total costs
Dengue	2013	9		1,863 €		582 €	2,445 €
Dengue	2014	11		2,277 €		704 €	2,981 €
Dengue	2014	4	16	828 €	7,100 €	1,280 €	9,208 €
Dengue	2014	5		1,035 €		320 €	1,355 €
Chikungunya	2014	6		1,242 €		384 €	1,626 €
Dengue	2015	1		207 €		63 €	270 €
Dengue	2015	7		1,449 €		439 €	1,888 €
Chikungunya	2016	2		414 €		124 €	538 €
Dengue	2016	4		828 €		248 €	1,076 €
Chikungunya	2016	5		1,035 €		310 €	1,345 €
Zika	2016	7		1,449 €		435 €	1,884 €
Dengue	2017	11		2,277 €		690 €	2,967 €
Zika	2017		8	0 €	4,600 €	502 €	5,102 €

* Intensive Care Unit

On the other hand, in order to evaluate a proxy estimate of the health burden of mosquito species in public health we also used the WNV costs induced by other mosquito species. Specifically, we present in Table 2 the medical costs of the 2010 WNV outbreak in Central Macedonia, Greece and their associated public health prevention and control strategies’ costs [24,25]. In this epidemic, which occured mainly in this Region, a total of 260 cases were hospitalized during the first year. In the following three years of transmission the number of hospitalized cases fell to 30, 18 and 22 (Table 2). It should be underlined that enhanced surveillance and control measures, were implemented during the first year of the outbreak and particularly during the peak months of transmission (from June to October).

**Table 2:** Cost of illness for WNV outbreak in Central Macedonia (2010–2013).

	2010	2011	2012	2013
Public prevention costs	6,300,000 €	4,600,000 €	4,002,000 €	3,052,000 €
Hospitalised cases	260	30	18	22
Cases treated in ICU*	25	2	1	2
Hospitalization costs	524,576 €	74,070 €	44,878 €	38,916 €
Hospitalization costs in ICU	162,300 €	14,200 €	7,100 €	20,700 €
**Total medical costs**	**686,875 €**	**88,270 €**	**51,978 €**	**59,616 €**
Productivity losses	229,553 €	30,636 €	19,047 €	17,195 €
**Total cost of illness** **(medical cost and productivity losses)**	**916,429 €**	**118,905 €**	**71,025 €**	**76,811 €**
**Cost of illness per case**	**3,524 €** **[563, 30,769]**	**3,963 €** **[280, 37,515]**	**3,946 €** **[563, 28,309]**	**3,491 €** **[563, 26,503]**

* Intensive care units

It might seem that costs associated with WNV (which is spread mainly by *Culex* mosquitoes) may be a rather poor approximation to health impacts of the Asian tiger mosquito. However, even though the costs for the WNV outbreak cannot be directly attributed to IMS, they represent an up-to-date indicator of regular public expenses incurred against the spread of mosquito-borne diseases in Greece. This indicator is directly comparable to other relevant estimates in South Europe [26], thus enabling better adjustment for country-level effects (variation) in the cost-of-illness assessment. In addition, it is also interesting to note that the annual regional surveillance program for arbovirsuses in the Emilia Romagna Region of Italy [27] is designed for the common surveillance of various vector borne diseases such as WNV, chikungunya and dengue, highlighting thus the importance of applying integrated approaches against all mosquito-borne diseases.

The estimation of health impact costs, (Tables 1 and 2) comprising medical costs and productivity losses, were based on the cost-of-illness (COI) analysis [28] in which the burden of a disease on society is estimated in financial terms using both direct and indirect measures. Direct costs consist mainly of medical care, both inpatient and outpatient, and are estimated using market prices. According to the National DRG (Diagnosis Related Groups) Indicators published in the 3054/18-11-2012 Official Government Gazette of the Hellenic Parliament, the average daily hospital care cost in Greek public hospitals is approximately 207€/day; this was multiplied by the total inpatient care days.

In addition, indirect costs represent the loss of productivity due to morbidity. These costs were estimated only for earnings lost during the reported days of sickness among people older than 18 years of age; the value of a lost working day was then multiplied by the total number of sick-days. The cost of a lost working day for people in the 18 to 65 years age range was calculated according to the per capita net income equivalent for the reference years (2011–2013) [29], divided by 220 working days. For people aged 65 years and over, the cost of a lost working day was calculated from the country’s median hourly earnings [30] for 2010, adjusted for inflation by the Consumer Price Index and then multiplied by 8 working hours. Due to lack of data on the age of patients, productivity losses for the imported cases of chikungunya, dengue and Zika virus, were calculated based from the median hourly earnings [30] for 2014, adjusted for inflation by the Consumer Price Index for each indicative year and then multiplied by 8 working hours.

### Estimation of benefit levels from improved mosquito control programs

We attempted to elicit household preferences for controlling IMS through a choice experiment approach [21]. Specifically, this stated preference method was implemented in order to examine household preferences regarding various attributes of mosquito control programs in relation to mosquito impacts (i.e. in order to assess the influence of these attributes in choosing a program). The initial selection of attributes was based on feedback from experts and on previous relevant studies. These attributes and their levels were then reduced to only those that were found to have a clear relationship with mosquito control programs, and this relationship was articulated in operational terms easily comprehended by citizens.

It should be noted that the control of mosquitoes is mainly carried out through annual activities which include monitoring and surveillance of the mosquito larvae population, implementation of larvicidal, adulticidal and surface residual ground treatments, and application of larvicidal and small scale adulticidal treatments by aerial spraying. On the other hand, controlling the Asian tiger mosquito calls for a more complex management plan and coordinated actions which have only recently been designed by the LIFE CONOPS research initiative (http://www.conops.gr/management-plan-for-aedes-albopictus-in-greece/?lang=en). The actions in this plan include (among others): standardized quantitative monitoring by special ovitraps, recording of mosquito population density data, involvement of the local population in the control campaign in private areas, residual door-to-door control interventions and use of larvicides in the road drains of public areas throughout the whole breeding season. The control methods and management plans according to the type of mosquitoes were described to all respondents at the beginning of the interview [21].

When selecting the attributes, two main categories of benefits that may be derived from improved mosquito control programs were identified: less nuisance and reduced risks to health. Another distinction was drawn between benefits from controlling native mosquitoes (principally of the *Culex* and *Anopheles* genera) and benefits from controlling invasive mosquitoes (the Asian tiger mosquito). For this purpose, two health risk attributes were used: (a) one related to the health risks that are mainly associated with native mosquitoes, such as WNV, and (b) another one related to the health risks due only to the Asian tiger mosquito (such as chikungunya fever). The nuisance attributes were likewise separated into: (a) nuisance during the day-time, which is a problem caused mainly by the Asian tiger mosquito, an “aggressive day-time biting mosquito” [31], and (b) nuisance at night, mainly associated with the native mosquito species. A cost attribute was included in order to elicit welfare effects, as determined by individuals’ preferences between alternative mosquito control programs.

Interviews were conducted from mid-June 2015 to the end of October 2015 in several districts within the Athens Metropolitan Area selected in order to represent the socioeconomic diversity of the city and the different degrees of exposure to the mosquito problem (either the Asian tiger mosquito or the native species). The survey was administered in face-to-face interviews by three trained interviewers and the average duration of each interview was 15 minutes. Although it was not possible to draw a strictly random sample from a sampling frame, the sample achieved a high degree of representativeness concerning geographical location and it was stratified based on location, sex and age (according to the 2011 Census). A total of 495 completed interviews were collected.

### Qualitative survey of experts on the effectiveness of mosquito control programs

The socioeconomic evaluation of the mosquito control strategies was enhanced by conducting a survey of experts on the issue of the overall mosquito problem. This survey was designed to evaluate the socioeconomic impacts of the mosquito control plans by interviewing key stakeholders, public policy makers, medical practitioners, public health experts and regional administrators. The questions were formulated in order to evaluate the results of the preceding studies (especially the choice experiment) and provide qualitative evaluation of specific policy-related decisions (ecosystem services, adequacy of control programs, etc.). The questionnaire was distributed to a pool of 100 experts all over the country, selected on the basis of their experience and involvement in the design and implementation of mosquito control strategies. The survey was conducted through telephone interviews from May 2016 to May 2017 in collaboration with a member staff of the Ministry of Health and a total of 59 responses were collected.

### Web-based survey on the qualitative impacts of the Asian tiger mosquito in Greek households

Apart from the survey in Athens, another questionnaire was also designed for a nationwide web-based survey aimed at eliciting citizens’ opinions regarding certain socioeconomic aspects of the mosquito problem. Its particular focus was to examine and then to validate at the national level a set of parameters related to: a) private prevention costs for IMS, and b) individual preferences among various mosquito control programs. The questionnaire was distributed through a popular meteorological data website (www.meteo.gr) with a high daily number of visitors [32]. For the purpose of our survey, a special banner appeared on the home page, from which visitors followed a link to the web survey. The banner appeared randomly to visitors, but a selection bias could arise due to (i) the non-representative nature of the internet population, and (ii) self-selection of participants (the `volunteer effect') which was possibly related to their interest in mosquito control. The first set of questions focused on the respondents’ knowledge of the Asian tiger mosquito. Subsequent questions concerned: (a) the current perceived level of nuisance during the day and separately at night, both rated using a 5-point Likert scale (nuisance impacts from the Asian tiger mosquito and from other species were estimated by attributing the nuisance during the morning and late afternoon hours to the former and the nuisance during the evening and night hours to native mosquito species), (b) the period (months/year) with significant mosquito nuisance, (c) the monthly household expenditure for private prevention measures, and (d) the main reasons for taking individual prevention measures (i.e. they had to choose between health risk reduction and nuisance reduction). The survey took place in September and October 2016 with a total of 1,220 responses from all over the country.

## Results

### Public control and prevention costs

According to national data published online on the governmental Greek Transparency Program Initiative (http://diavgeia.gov.gr), the average annual public mosquito control costs in the Athens Metropolitan area range from approximately 800,000 € to 1,330,000 € per year. This represents an average annual cost of about 0.6 € to 0.9 € per household. These programs consist mainly of adulticide and larvicide activities, mainly with the use of specific chemical larvicides currently available or undergoing the revision process in the EU [33], such as Diflubenzuron; these are designed for the control of *Culex* and *Anopheles* species and therefore target the elimination of their associated diseases (such as WNV). In other words, the implementation of these programs is not specifically tailored to the control of the Asian tiger mosquito and the prevention of chikungunya and dengue fever, even though Diflubenzuron also has high efficacy rates for the *Aedes* species [34]. It should be noted that some surveillance activities for the *Ae*. *albopictus* are currently implemented in most parts of Greece including the Athens metropolitan area [35], however, data are insufficient for calculating the cost of these as yet.

In contrast to Greece, some other European countries are implementing programs specifically aimed at the control of the Asian tiger mosquito, such as the *"Italian Plan of the Emilia-Romagna Regional Health Authority for the fight against the Asian tiger mosquito and the prevention of Chikungunya and Dengue fever"* [36]. The current Greek management plan differs from these chiefly in that because it is not focused on combating the Asian tiger mosquito, larvicide activities mainly take place in public spaces without considering specific urban (residential) areas with high breeding activity of *Aedes albopictus*. The lack of a regional or national plan aimed specifically at controlling the Asian tiger mosquito makes the measurement of the efficacy of larvicide activities against *Aedes albopictus* difficult.

What is more, due to high domestic breeding patterns of the *Aedes* species, information and communication activities can have a very high impact on the control of mosquito populations. According to recent estimates [36], the annual total expenditure for information activities in Emilia-Romagna during the years 2009–2011 ranged from 150,000 €/year to 0.6 mil €, significantly lower rate than the costs for regular anti-larval treatments which ranged from 3.6 to 4.4 million €/year. As previously noted, the annual integrated surveillance plan for arboviral diseases in Emilia Romagna is designed for the common surveillance of various vector-borne diseases such as WNV, chikungunya and dengue, while recent studies also emphasize the effectiveness of community participation also concerning the elimination of *Culex* species [37].

The overall public control and prevention costs associated with the WNV epidemic have already been presented in Table 2. Higher costs in the first year of application of measures are justified as a contingent response to the expansion of the outbreak. However, it appears that costs fell significantly during the following years. This could be also interpreted as a result of the epidemic being partially controlled; however, there are inadequate data within this study to support this argument.

### Medical costs and productivity losses for imported cases of dengue, chikungunya and Zika virus cases

According to the results Table 1, the average health cost for an imported case of Dengue was estimated to be 1,170 € for chikungunya, 2,774 € for dengue and almost 3,500 € for Zika virus. Even though the overall socioeconomic costs in the case of epidemic outbreaks for these diseases cannot be estimated with high precision (due to the limited number of disease cases), it is possible that in the scenario of future epidemics, disease complications could outweigh the present costs of treating the diagnosed imported cases.

### Medical costs and productivity losses for the recorded WNV cases

The total cost of illness (COI) in the first year of the WNV outbreak (2010) was estimated at about 900,000 € [20]. This includes the cost of hospitalization for 260 recorded WNV cases, 25 of whom needed further hospitalization in intensive care at an extra cost of about 160,000 €. The total COI in the following year was estimated to be nearly 120,000 € for the hospitalization of 30 cases (two of whom required treatment in intensive care units). Subsequently, 18 cases were recorded and treated in 2012 with only one case requiring intensive care. The total COI for this year amounted to 71,000 €. Finally, in 2013, 22 cases were diagnosed (two in intensive care) and the COI was correspondingly slightly higher at 77,000 € (Table 2).

### Results on the benefit levels of the choice experiment method

Even though it is very difficult to provide precise estimates of the total costs and the total social benefits of mosquito control programs, the results of our previous study [21] permit us to conclude that the benefits of mosquito control in terms of reduced nuisance and reduced health risks are likely to exceed the associated implementation costs.

Under our most conservative scenario (i.e. a medium prevention scenario, effective only against the native mosquito species), the estimated aggregate benefits from improved control programs can reach up to 11.2 million €/year, thus corresponding to a net benefit of 7.40 €/household/year (see Table 3). These results provide an order of magnitude estimate of the economic feasibility of improved mosquito control programs in the study area (Athens Metropolitan area). Specifically, the benefit-cost ratio of any program which is expected to achieve the selected target levels at a cost less than 13 times the cost of the current mosquito control program (800,000 €/year) will be greater than one (i.e. would be economically profitable). This cost could further increase (up to 31.3 €/household/year) if a high prevention scenario, effective against all mosquito species, were implemented. On the other hand, the expected added value of taking measures not only against native but also against the Asian tiger mosquito was found to be substantial, representing on average an additional benefit of about 15€/household/year [21]. As shown in Table 3, this benefit can be attributed mainly to the high health risks posed by the introduction of new invasive species into the study area.

**Table 3:** Households’ benefits as estimated for various prevention scenarios (results from the survey conducted in Athens [21]).

	Benefits (€/household/year) and their confidence intervals				
	West Nile virus risk reduction	Tiger mosquito health risk reduction	Night nuisance reduction	Day nuisance reduction	Total benefits
High prevention scenario against all mosquito species	10.38 [6.6, 13.8]	13.86 [10.5, 17.2]	5.76 [3.6, 7.9]	1.3 [-0.9, 3.9]	**31.30** **[19.8, 42.8]**
Medium prevention scenario against all mosquito species	5.19 [3.3, 6.9]	13.86 [10.5, 17.2]	2.21 [1.4, 3.0]	0.10 [-0.1, 0.3]	**21.36** **[15.1, 27.4]**
High prevention scenario against native mosquito species	10.38 [6.6, 13.8]	-	5.76 [3.6, 7.9]	-	**16.14** **[10.2, 21.7]**
Medium prevention scenario against native mosquito species	5.19 [3.3, 6.9]	-	2.21 [1.4, 3.0]	-	**7.40** **[4.7, 9.9]**

### Results of the survey of experts

In the survey of experts, 48% of the respondents considered the financial budget allocated to control programs to be adequate for confronting the problem while 34% suggested that an increase in public spending would be necessary. In addition, experts judge that the current control programs achieve balance between cost and effectiveness in their design and implementation. With respect to the potential negative impact of prevention measures on relevant ecosystem services, 65% of the experts stated that there are no (significant) negative impacts from these measures. Regarding the means of obtaining extra funds for supporting mosquito management, experts indicated that: (a) a redistribution of public resources would be necessary, (b) a reallocation of funds within national and regional budgets could improve the financing of mosquito control programs, and that (c) a financial contribution by citizens is equally important for the confrontation of the problem.

It should be noted that the Asian tiger mosquito can exploit water containers in private apartments for its breeding. Therefore, according to the experts, private prevention activities could contribute significantly to the reduction of the problem at a much lower cost, especially if supported by public information activities which, as shown in the case of Emilia Romagna in Italy, could be more cost-effective. Lastly, regarding the prioritization of the objectives of future control programs (Table 4), experts stated that the health impacts should be considered as the primary objective of these programs. Specifically, they consider the health threats of native and invasive mosquito-borne diseases as almost equally important, whereas they treat nuisance from mosquito species as a less important impact factor.

**Table 4:** Rating of the objectives of mosquito control programs based on the answers of 59 experts.

	Reduction of mosquito-borne disease risk		Reduction of nuisance	
	from native species	from invasive species	from native species	from invasive species
**Highly important**	**63%**	29%	4%	0%
**Important**	32%	**56%**	7%	3%
**Neutral**	3%	7%	**64%**	12%
**Less important**	2%	3%	20%	**61%**
**Not important**	0%	3%	5%	24%
**N/A**	-	2%	-	-

### Results of the web survey of private citizens

In the web survey of private citizens, 83% of respondents stated that the current prevention and control measures are insufficient or inadequate for dealing with the mosquito problems and therefore there is a need for further measures to be taken. The average private prevention costs of the sample were approximately 16€ per month in the period when mosquitoes are active, which amount to about 100 €/year. There was significant regional variation in these estimates, ranging from below 80 € (e.g. Region Thessaly and Region of North Aegean) to over 125 € (e.g. Region of Eastern Macedonia and Thrace, and Region of Central Greece). This variation may be an indirect indicator of the magnitude of the mosquito problem, which is strongly associated with the nuisance conditions in each area. It should be also noted that this revealed behavior concerning prevention costs can be used as a lower-bound proxy of individuals’ potential benefits from improved control measures in each region.

The results from this survey concerning the preferences of individuals for the diverse mosquito control programs are shown in Table 5. Concerning the main targets of these measures (Table 5), health impacts were considered to be more important than nuisance impacts, confirming the findings of previous surveys in Greece [20,21].

**Table 5:** Rating of the objectives of mosquito control programs’ (web-survey results) based on the answers of 1220 citizens.

	Reduction of mosquito-borne disease		Reduction of nuisance	
	From native species	From invasive species	From native species	From invasive species
**Highly important**	**73.2%**	**76.7%**	**47.1%**	**39.5%**
**Important**	19.1%	15.9%	32.3%	25.3%
**Neutral**	5.4%	5.6%	15.7%	20.2%
**Less important**	1.6%	1.2%	4.0%	10.3%
**Non important**	0.7%	0.6%	0.9%	4.7%

Furthermore, as in the other two studies, health risks from invasive species were considered to be a serious threat. Therefore, both group, (experts and citizens), appear to rate the health risks higher compared to the nuisance and cost factors of mosquito control programs. Finally, an important finding of this survey was that citizens seem to be aware of the environmental consequences of mosquito control measures. In particular, about 74% of respondents stated their disagreement with measures that may potentially affect the physical environment and the ecosystems.

## Discussion

The present paper aims at an overview of the socioeconomic aspects of the problem of invasive mosquitoes as recorded by the main interest groups in society (citizens and experts). It provides substantive indicators regarding the citizens’ perceived benefit derived from the implementation of improved mosquito control programs, as well as experts’ evaluation of the socioeconomic effectiveness of current and future programs for controlling the problem of invasive mosquitoes. In contrast to other studies [17,38,39] findings from both perspectives show a higher priority for the prevention and reduction of health risks as opposed to nuisance control.

Furthermore, based on the results of the survey conducted in Athens, citizens are willing to pay a considerable premium for effective protection against the spread of unfamiliar diseases, thus implying a risk-averting behavior against invasive mosquito threats. In other words, citizens are willing to pay today for improved control programs that will be able to eliminate potential future impacts and risks. The fact that climate change trends may worsen the mosquito problem and increase the risks of the transmission of new diseases (such as Zika virus) is likely to provide ever increasing potential individual and social benefits from implementing more efficient mosquito control management plans in the coming years [40].

The cost estimates extracted from the current study allow for a comparison with recent similar estimates in Southern Europe. According to our findings the costs of public mosquito control programs range from approximately 0.6 € to 0.9 € per household in Athens, while the public costs of informed arbovirus plans in the Region of Emilia Romagna in Italy reach almost 1.2 € per household [36]. Using the Purchasing Power Parity Index [41] this figure translates to an equivalent of 1.04 €/year per household in Athens, indicating that a small per capita increase in public costs could justify the design and implementation of more targeted programs in Greece, in terms of perceived citizens’ benefits levels as already presented in the current analysis.

What is more, a recent study estimated that the implementation of public intervention strategies against the spread of *Aedes* related arboviruses in Italy since 2007 may have saved up to 13.5 million € indicating the cost-effectiveness of these interventions both from an economic and a health perspective [23]. It should be noted that our analysis indicated an annual benefit of up to 11 million € from the implementation of optimal mosquito control programs in the Athens area intended to achieve public health targets similar to those of Italy.

With regard to health impact costs, the medical costs for an average cost of illness of an imported chikungunya virus case in Italy reaches approximately 3,500 € [23], while the average in Greece, based on our limited sample of imported disease cases estimated to be about 1,100 €. The average cost of illness for all three types of imported diseases in Greece (chikungunya, dengue, Zika virus) was found to be approximately 2,500 €. According to another recent study in La Reunion [22], the mean cost of illness per inpatient case of chikungunya reached approximately 2,000 €. Estimates of the cost of illness of recent WNV epidemics indicate a cost of about 3,500 € per case in Greece, while in Italy cost data are only available for the mean cost of illness for a WNV case with neuroinvasive complications (WNND), which reaches approximately 15,000 € per case [26].

The above estimates offer an important range of socioeconomic figures relevant to mosquito-related diseases in Southern Europe which could act as significant indicators for evaluating the societal benefits of integrated public control programs against the spread of arbovirus diseases under turbulent climatic and societal conditions.

The establishment of invasive species is usually associated with increased economic costs. For example, a study in the USA [42] estimated environmental damages and losses of almost $120 billion per year. According to a European Commission Impact Assessment [43], Invasive Alien Species are estimated to have cost the EU at least €12 billion/year over the past 20 years and the damage costs continue to increase. It is predicted that, due to the trends in climate change, the invasive mosquito problem will intensify in the immediate future [44]. Therefore, the evaluation of the socioeconomic costs of invasive mosquitoes is a vital but highly challenging task made even more complex by changing climatic conditions, as well as by globalization and urbanization trends that may call for the adoption of multi-disciplinary and more holistic approaches in order to evaluate the effectiveness of the expenses incurred in improving public health and social welfare [45].

## Supporting information

S1 Survey Questionnaire Web-based survey questionnaire. (PDF)

S2 Survey Questionnaire Expert’s survey questionnaire. (PDF)

S1 Fig Choice experiment card. (PDF)
