# Supplementary material for: On lifestyle trends, health and mosquitoes: Formulating welfare levels for control of the Asian tiger mosquito in Greece
Source: PLoS Negl Trop Dis. 2019 Jun 4;13(6):e0007467. doi: 10.1371/journal.pntd.0007467 (PMC6568418; doi:10.1371/journal.pntd.0007467)
Supplement: S1 Survey Questionnaire — (PDF) [file pntd.0007467.s001.pdf]

LIFECONOPS
“Development and Demonstration of management plans against the climate-enhanced invasive mosquitoes in Southern Europe” (LIFE12ENV/GR/0046)

ΜΠΕΝΑΚΕΙΟ ΦΥΤΟΠΑΘΟΛΟΓΙΚΟ ΙΝΣΤΙΤΟΥΤΟ	ΙΝΣΤΙΤΟΥΤΟ ΑΣΤΙΚΟΥ ΠΕΡΙΒΑΛΛΟΝΤΟΣ &ΑΝΘΡΩΠΙΝΟΥΔ ΥΝΑΜΙΚΟΥ	Mete

Web questionnaire for the appraisal of the consequences of the Asian tiger mosquito in Greece

The present questionnaire is part of the LIFE CONOPS project which studies the impact of invasive mosquito species such as the Asian tiger mosquito (Aedes albopictus) in Greece and Italy. More specifically, it aims at developing integrated plans for the management of mosquito control, in order to ensure the control of their spread and establishment throughout Europe.

The LIFE CONOPS project (LIFE12 ENV / GR / 000466) is 50% co-funded by the European LIFE + Environment Policy and Governance program. More information can be found on the project website, www.conops.gr.

General Information regarding the Asian tiger mosquito

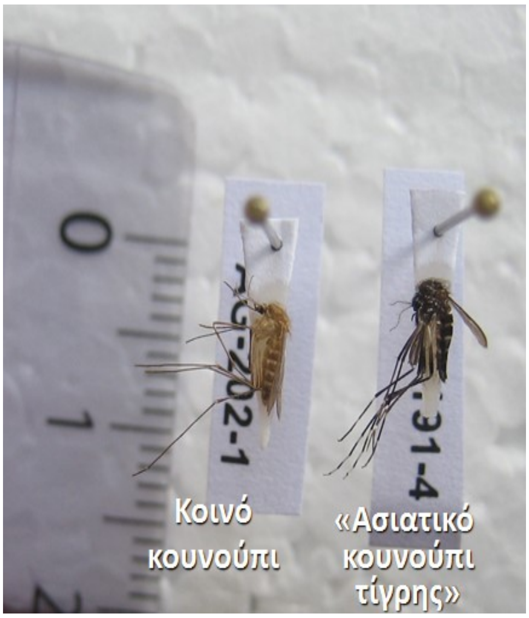

The Asian tiger mosquito is a regular mosquito with a body length similar to that of the common mosquito (5-6 mm). Its main characteristic is the black and white coloration of the body and the fact that females are aggressive and usually bite during the day with their maximum activity observed early in the morning (06:00 to 08:00) and late afternoon (16:00 to 18:00).

The public health risks of the Asian tiger mosquito

From a public health point of view, the Asian tiger mosquito is of great importance as it can transmit numerous infectious diseases such as Dengue fever, Zika and Chikingunya viruses. On the contrary, it does not appear to transmit other mosquito-related

diseases such as malaria and West Nile virus.

Irrespective of disease transmission capacity, its health significance lies also in the intense disturbance caused by its bites, particularly in urban areas where it is difficult to cope due to the numerous breeding sites. The nuisance it causes is intense and often the sensitivity reaction on the skin of the victims is evident causing redness, itching or even rashes. On the project's website you can find more related information, www.conops.gr.

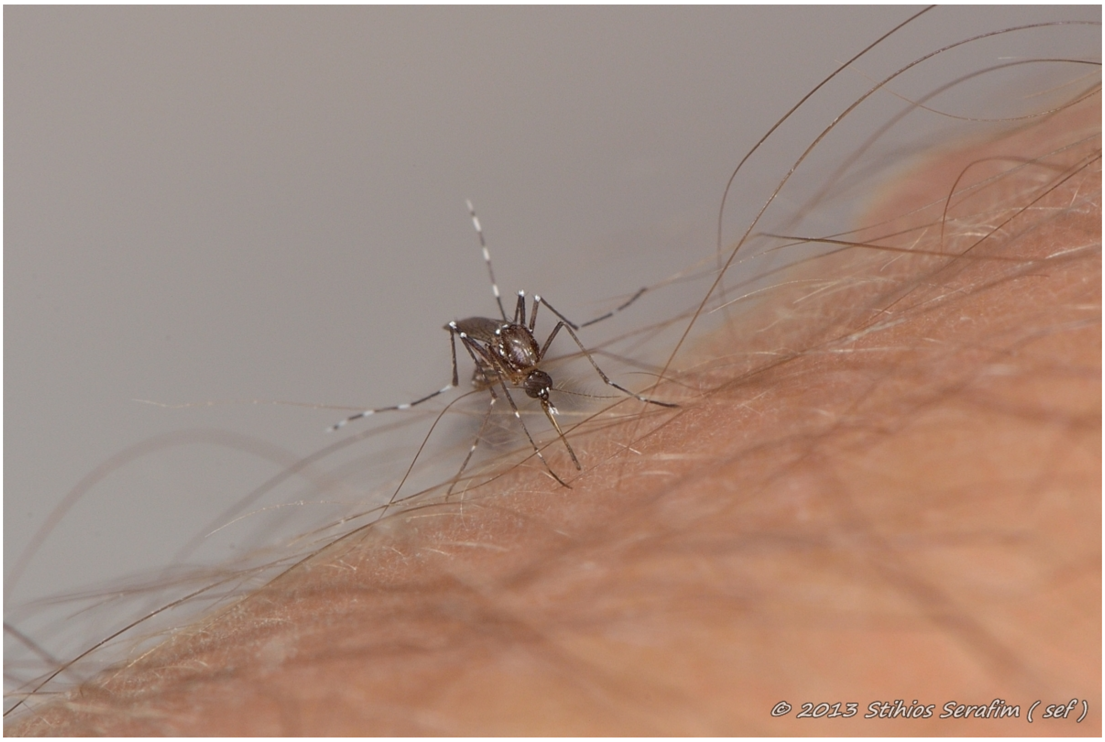

Questionnaire

Question 1
Were you aware of the Asian tiger mosquito before reading the introduction of the present questionnaire?

YES ☐ NO ☐

Question 2
Are you aware whether the Asian tiger mosquito is active in your residence area?

YES ☐ NO ☐

Question 3
How you would characterize the nuisance level by mosquitoes in your residence area?

i) during nighttime?

Unbearable ☐ Intense ☐ Medium ☐ Small ☐ Inexistent ☐

ii) during the first morning hours and late afternoon?

Unbearable ☐ Intense ☐ Medium ☐ Small ☐ Inexistent ☐

Question 4
Do you consider the mosquito problem intensified in your residence area in comparison with last year?

YES ☐

NO ☐

If YES, for which reason do you think it has intensified?
...............................(open answer)

Question 5
During which months do you face an intense mosquito problem in order to make use of specific private mosquito control measures? (mosquito repellents, mosquito nets, etc)

Initial Month:..........
Last Month:............

Question 6
In the afore mentioned month period, how much money do you spend on an average monthly basis, in your household for the confrontation of the mosquito problem?

.........................(open answer)

Question 7
Which is the main reason for taking special mosquito control measures?

The reduction of mosquito nuisance levels ☐
The reduction of health risks attributed to the presence of mosquitoes ☐

Question 8

How would you rate the following sentences (1= totally agree, 5= totally disagree):

- ☐ I consider the overall mosquito problem as serious but I think that more funds should be allocated to other sectors and problems
- ☐ I consider the overall mosquito problem as important but I am not aware of the exact negative consequences of the public mosquito control measures to the rest of the ecosystem
- ☐ I consider that further mosquito control measures should be taken regardless of the negative side-effects in the rest of the ecosystems
- ☐ I consider that the correct application of public control measures will not bear negative side-effects on the rest of the ecosystems
- ☐ I do not consider the overall mosquito problem as important

Question 9

How would you rate the public control measures in regards to **their adequateness in controlling the overall mosquito problem?**
(Please rate from 1-5, where **1 = non adequate**, **5= most adequate**)

- ☐ 1
- ☐ 2
- ☐ 3
- ☐ 4
- ☐ 5

Question 10

Could you prioritize the public mosquito control measures in regards to their public health targets ?
(Please rate from 1= the less important to 5= the most important)

- ☐ Reducing disease risk attributed to culex and other endemic species (e.g West Nile Virus, Malaria)
- ☐ Reduction of disease risk attributed to invasive mosquito species (Zika virus, Chikungunya, Dengue)
- ☐ Reducing nuisance in the evening hours
- ☐ Reducing nuisance in the morning and late afternoon hours
- ☐ Implementation Cost

Question 11

Has any of your household members ever become ill from a mosquito borne disease? (e.g West Nile Virus, Malaria)

YES ☐ NO ☐

If yes, from which disease?..

Personal Information:
Residence area (Address, Municipality, Region):
Household Members:
Age:
Contact details (phone number or email):
