# Supplementary material for: On lifestyle trends, health and mosquitoes: Formulating welfare levels for control of the Asian tiger mosquito in Greece
Source: PLoS Negl Trop Dis. 2019 Jun 4;13(6):e0007467. doi: 10.1371/journal.pntd.0007467 (PMC6568418; doi:10.1371/journal.pntd.0007467)
Supplement: S2 Survey Questionnaire — (PDF) [file pntd.0007467.s002.pdf]

LIFE CONOPS QUESTIONNAIRE ON THE APPRAISAL OF SOCIOECONOMIC IMPACTS OF MANAGEMENT PLANS BY STAKEHOLDERS AND POLICY MAKERS

1. There is (or you think there is) any problem of mosquitoes in your specific region and how would you rate it?

- ☐ There is no problem
- ☐ There is a problem but it is minor
- ☐ The problem is only in certain regions (eg. In rural, urban, natural ecosystems.)
- ☐ There is severe problem in both urban and rural areas

2. Do you know if in your area, in recent years, the "tiger mosquito" has settled, or other new types of mosquitoes (probably invasive) which did not exist before (over a decade)?

- ☐ Is installed
- ☐ Not installed
- ☐ Do not know

3. Do you know if there are recorded outbreaks of diseases transmitted by mosquitoes in your region (eg. Malaria, West Nile virus, Dengue virus, Chikungunya, Zika virus)? The cases were imported or indigenous (ie. The transmission took place in our country)?

- ☐ Outbreaks have been recorded
- ☐ No cases have been recorded
- ☐ Do not know

If yes, which, of these diseases were native and / or imported (eg. Imported cases of malaria and West Nile Virus),..

4. How have you formed the image you described about the problem of mosquitoes in your area? (Multiple responses accepted)

- ☐ Briefing on reports or actions scientific bodies or governmental institutions.
- ☐ Information from reports or presentations to media.

- ☐ Personal considerations
- ☐ Complaints of citizens in the region
- ☐ Outbreaks have been recorded (native - imported)

5. Do you know if the municipality, Region or Regional Unit, or the office with which you are involved, is engaged in addressing the problem of mosquitoes in your area?

• Involved / actively

• Not Involved

• Do not know

6. You know the kind of actions your service involved? (Multiple responses accepted)

Mosquito Control

- ☐ conduct spraying with the same instruments,
- ☐ project tender for implementation by contractors,
- ☐ participation in other actions (eg. Channel cleaning, reduce outbreaks, etc.)
- ☐ Monitor and control the course of fighting work performed by a contractor (an independent body)

Informing the public

- ☐ organizing information meetings / lectures,
- ☐ TV spots,
- ☐ Entries press, publication and distribution of brochures
- ☐ Actions in schools, or other groups.

Health care - Medical Actions

- ☐ actively search cases,
- ☐ passive search cases,
- ☐ Display monitoring network traffic or diseases transmitted by mosquitoes,
- ☐ patient care,

☐ care to avoid spreading the relative environment, by blood transfusions, etc.).

☐ Other actions ....

7. Which of these 3 is regarded as most important (please rate the boxes from 1 = most important to 3 = less important)

• mosquito control actions

• Information measures for the problem of mosquitoes

• Health Care - Medical Actions

8. Of the three categories of actions what you see as the most important sub-actions in any class action? (Please rate the boxes from 1 = most important to 3 = less important)

A. Mosquito control actions

☐ conduct spraying with the same instruments,

☐ project tender for implementation by contractors,

☐ participation in other actions (eg. Channel cleaning, reduce outbreaks, etc.)

II. Information measures for the problem of mosquitoes

☐ organizing information meetings / lectures, actions in schools, KAPI, or other groups

☐ TV spots,

☐ Entries press, publication and distribution of brochures

III. Health Care - Medical Actions

☐ active cases search, passive search cases,

☐ Display monitoring network traffic or diseases transmitted by mosquitoes,

☐ care patients, care to prevent the spread relative to the environment, by blood transfusions, etc.

9. You as a person / agency / institution / agency are involved in the programs or actions to address the problem of mosquitoes in your area of competence? If so, how and to what extent?

• YES

• NO

If so, how and to what extent: ....

10. Do you know the amount of funding spent on programs of those activities in your area. Do you have data or estimates about the amount per action (or even if funding is only for sprays or other actions)?

Amount of funding / year...

amounts per
Action:...
....
....
....

11. Are the expected results from the programs or actions achieved, in order to address the problem of mosquitoes in your area of competence?

(Please rate on a scale from 1 = complete success to 5 = almost no successful)

- ☐ 1
- ☐ 2
- ☐ 3
- ☐ 4
- ☐ 5

12. Do you know or believe that there are problems in the implementation of programs that have an impact on their success? (Multiple responses accepted)

- ☐ Bureaucratic procedures.
- ☐ Ensure funding and permanent character.
- ☐ Operational problems
- ☐ Absence or inadequate supervision and evaluation of programs
- ☐ Reduced public response
- ☐ Inability to access private areas
- ☐ Other problems ....

13. Do you consider that the amount spent is sufficient? You think it is necessary increase / (or decrease) of that amount?

- ☐ Sufficient
- ☐ Increase
- ☐ Decrease

14. Do you think it is necessary to have a permanent provision and funding to deal with mosquito control or should they take place only when these problems occur (eg. Cases, nuisance above tolerable levels, outbreaks etc.)? Note that in cases not timely applied directly address the problems can be very difficult and / or impossible.

- ☐ YES
- ☐ NO

15. How do you think they could secure additional financial resources or means to improve programs or actions? (Possibly multiple responses accepted)

- ☐ Reallocation of State Budget
- ☐ Redistribution of Resources by Region or the municipality.
- ☐ Transfer funds from other Municipalities / Regions actions etc.
- ☐ Imposition of special fees to citizens
- ☐ Obligation of individuals to take some action / sprays in private spaces.

16. How would you prioritize the necessity of programs in relation to the objectives that serve? (Please rate the boxes from 1 = most important to 5 = least important)

- ☐ Reducing wrecks of disease risk
- ☐ Reduction of disease risk from new species
- ☐ Reducing nuisance in the evening
- ☐ Reducing nuisance in the morning
- ☐ Implementation Cost

17. Please rate from 1 to 5 (1 = max to 5 = minimum) the extent to which you believe that those involved by municipalities (or depending on the region or Regional Unit) or the service you belong to have the necessary scientific training / expertise / experience to diagnose the problem, prioritizing needs, designing programs and activities, training notices, monitoring and evaluation of programs in your area?

- ☐ 1
- ☐ 2
- ☐ 3
- ☐ 4
- ☐ 5

18. Do you believe that a) it would be useful / necessary to get by municipalities (or depending on the region or Regional Unit) or the Service you belong to the necessary scientific background and expertise in order to achieve better results? or b) it is preferable to assigning specialized external scientific institutions (public or private).

- ☐ It would be useful
- ☐ It would be useful
- ☐ It is preferable to assigning specialized external scientific bodies

19. Do you consider that the design of programs and actions, training notices, supervision and evaluation of the programs should be central to the whole country or stay at local level - and to what (eg. Regional Level, Regional Unity municipality, etc.).

- ☐ Main Level
- ☐ Local Level (specify) ....
