# Supplementary material for: On lifestyle trends, health and mosquitoes: Formulating welfare levels for control of the Asian tiger mosquito in Greece
Source: PLoS Negl Trop Dis. 2019 Jun 4;13(6):e0007467. doi: 10.1371/journal.pntd.0007467 (PMC6568418; doi:10.1371/journal.pntd.0007467)
Supplement: S1 Fig — (PDF) [file pntd.0007467.s003.pdf]

Assuming that the following mosquito control programs are the only choices you can made, which one do you choose?			
	**Option A**	**Option B**	**Option C (Status quo)**
Risk level of a WNV outbreak	Average risk	High risk	Neither Option A, nor Option B<br><br>Current situation (i.e. current mosquito control program)
Further public health actions to reduce the Tiger Mosquito health risks	YES	NO	
Nuisance level of mosquitoes during day time	No improvement	Improved level – Low Nuisance	
Nuisance level of mosquitoes during night time	Improved level – Low Nuisance	Improved level – No Nuisance	
Expected bi-monthly cost	10€	15€	0 €
**Choice**			

Source: Bithas, K., Latinopoulos, D., Kolimenakis, A. and Richardson, C., 2018. Social benefits from controlling invasive Asian tiger and native mosquitoes: a stated preference study in Athens, Greece. Ecological Economics, 145, pp.46-56.
